# 6a-Nitro-6-(2,2,7,7-tetra­methyl­tetra­hydro-3a*H*-bis­[1,3]dioxolo[4,5-*b*:4′,5′-*d*]pyran-5-yl)-6a,6b,7,8,9,11a-hexa­hydro-6*H*-spiro­[chromeno[3,4-*a*]pyrrolizine-11,11′-indeno­[1,2-*b*]quinoxaline]

**DOI:** 10.1107/S1600536813032467

**Published:** 2013-12-14

**Authors:** T. Anuradha, J. Naga Siva Rao, P. R. Seshadri, R. Raghunathan

**Affiliations:** a Post Graduate & Research Department of Physics, Agurchand Manmull Jain College, Chennai 600 114, India; b Department of Organic Chemistry, University of Madras, Guindy Campus, Chennai 600 025, India

## Abstract

In the title compound, C_39_H_38_N_4_O_8_, the quinoxaline and indene subunits are essentially planar, with maximum deviations of 0.071 (2) and 0.009 (2) Å, respectively. The indeno­quinoxaline system forms a dihedral angle of 72.81 (3)° with the chromenopyrrolizine system. The two dioxolane rings, as well as the pyran ring of the chromeno group and the terminal pyrrolizine, each adopt an envelope conformation with O and C as flap atoms. The central pyrrolizine ring adopts a twisted conformation. Intra­molecular C—H⋯O and C—H⋯N hydrogen bonds occur. The crystal structure exhibits C—H⋯O hydrogen bonds, and is further stablized by C—H⋯π inter­actions, forming a two-dimensional network along the *bc* plane.

## Related literature   

For some spiro compounds of biological importance, see: Kobayashi *et al.* (1991 ▶); James *et al.* (1991 ▶). For the pharmaceutical importance of quinoxaline derivatives, see: Seitz *et al.* (2002 ▶); He *et al.* (2003 ▶). For conformation analysis, see: Cremer & Pople (1975 ▶).
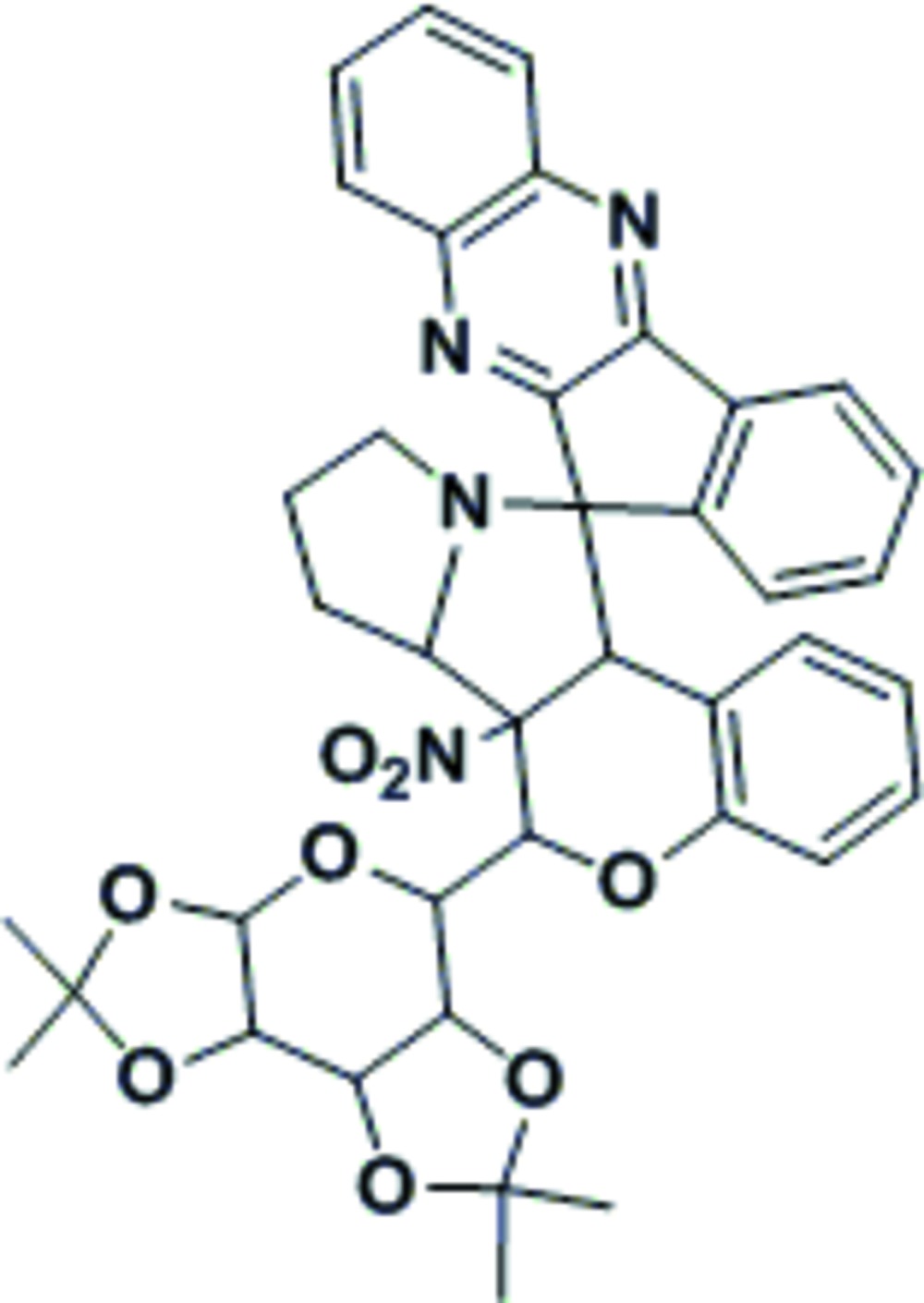


## Experimental   

### 

#### Crystal data  

C_39_H_38_N_4_O_8_
*M* *_r_* = 690.73
Orthorhombic, 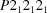

*a* = 11.3150 (9) Å
*b* = 15.629 (2) Å
*c* = 19.419 (2) Å
*V* = 3434.0 (6) Å^3^
*Z* = 4
Mo *K*α radiation
μ = 0.09 mm^−1^
*T* = 293 K
0.20 × 0.15 × 0.10 mm

#### Data collection  

Bruker Kappa APEXII CCD diffractometer
Absorption correction: multi-scan (*SADABS*; Bruker, 2004 ▶) *T*_min_ = 0.981, *T*_max_ = 0.991
15819 measured reflections
6671 independent reflections
3789 reflections with *I* > 2σ(*I*)
*R*_int_ = 0.059

#### Refinement  

*R*[*F* ^2^ > 2σ(*F* ^2^)] = 0.050
*wR*(*F* ^2^) = 0.124
*S* = 0.97
6671 reflections
460 parameters
H-atom parameters constrained
Δρ_max_ = 0.17 e Å^−3^
Δρ_min_ = −0.20 e Å^−3^
Absolute structure: Flack (1983 ▶), 2933 Friedel pairs
Absolute structure parameter: 0.30 (13)

### 

Data collection: *APEX2* (Bruker, 2008 ▶); cell refinement: *SAINT* (Bruker, 2008 ▶); data reduction: *SAINT*; program(s) used to solve structure: *SHELXS97* (Sheldrick, 2008 ▶); program(s) used to refine structure: *SHELXL97* (Sheldrick, 2008 ▶); molecular graphics: *ORTEP-3 for Windows* (Farrugia, 2012 ▶); software used to prepare material for publication: *SHELXL97*, *PLATON* (Spek, 2009 ▶) and *publCIF* (Westrip, 2010 ▶).

## Supplementary Material

Crystal structure: contains datablock(s) I, New_Global_Publ_Block. DOI: 10.1107/S1600536813032467/im2440sup1.cif

Structure factors: contains datablock(s) I. DOI: 10.1107/S1600536813032467/im2440Isup2.hkl

Additional supporting information:  crystallographic information; 3D view; checkCIF report

## Figures and Tables

**Table 1:** Hydrogen-bond geometry (Å, °) *Cg*8 is the centroid of the N1/C7/C14/N2/C13/C8 ring.

*D*—H⋯*A*	*D*—H	H⋯*A*	*D*⋯*A*	*D*—H⋯*A*
C35—H35⋯O2^i^	0.98	2.41	3.390 (4)	177
C16—H16*A*⋯O3	0.97	2.59	3.237 (5)	124
C21—H21⋯N2	0.98	2.48	3.305 (4)	141
C30—H30⋯*Cg*8^ii^	0.98	2.93	3.895 (4)	168

Symmetry codes: (i) 
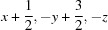
; (ii) 
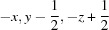
.

**Fig. 1.**
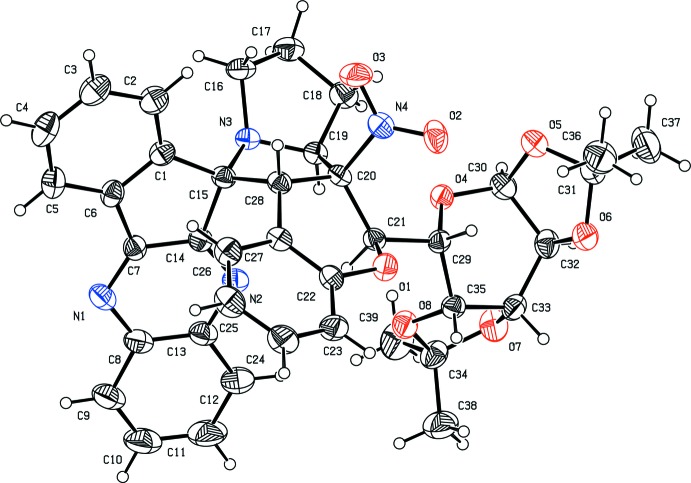
Molecular structure of the title compound showing 30% probability displacement ellipsoids. H atoms are shown as spheres of arbitrary radius.

**Fig. 2.**
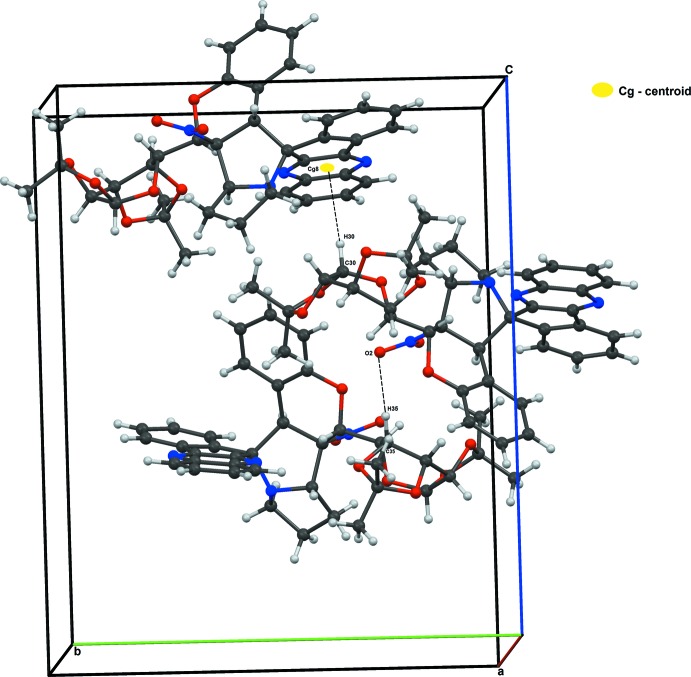
A view of packing of the molecules with hydrogen bonds (dashed lines).

## supplementary crystallographic information

### 1. Comment

Spiro compounds are a particular class of naturally occurring substances characterized by highly pronounced biological properties (Kobayashi *et al.*, 1991; James *et al.*, 1991). Quinoxaline derivatives are an important class of benzoheterocycles. They have found applications as anticancer, antiviral, and antibacterial agents (Seitz *et al.*, 2002; He *et al.*, 2003).

In the title compound, C_39_H_38_N_4_O_8_, the quinoxaline (C7—C14\N1\N2) and indene (C1—C7\C14\C15) subunits are essentially planar with maximum deviations of -0.071 (2) Å for C14 and -0.009 (1) Å for C9, respectively. The dihedral angle between the chromeno-pyrrolizine (C15—C28\N2\N3\O1) and indeno-quinoxaline (C—C15\N1\N2) systems is 72.81 (3)° showing their almost orthogonal arrangement relative to each other.

The two dioxolane rings (C30—C32\O5\O6,C33—C35\O7\O8) which are fused with a pyran ring adopt an envelope conformation with O6 and C34 atoms as the flap. Puckering parameters are q_2 _= 0.3089 (1) Å, Φ_2_ = 108.05 (2)° and q_2 _= 0.2905 (1) Å, Φ_2_ = 324.14 (2)°, respectively. The pyran ring (C20—C22\C27\C28\O1) of the chromeno group and the pyrrolizine ring (C16—C19\N3) also adopt envelope conformations with O1 and C16 atoms as the flap. Puckering parameters are q_2 _= 0.3895 (1) Å, q3 = -0.2566 Å, Φ_2_ = 235.74 (2)°, Θ = 123.38° for the pyran and q_2 _= 0.3781 (1) Å, Φ_2_ = 213.99 (4)° for the pyrrolizine system, respectively. The pyrrolizine ring fused with the pyran ring adopts a twisted conformation along N3—C15 with puckering parameters of q_2 _= 0.301 (1) Å, Φ_2_ = 200.75 (2)° (Cremer & Pople, 1975).

The structure is stabilized by an intermolecular C—H···O hydrogen bond and additional intramolecular C—H···O and C—H···N hydrogen bonds (Table 1). The crystal structure is further consolidated by C—H ···*Cg*8 interactions where *Cg*8 is the centroid of C7\C8\C13\C14\N1\N2 ring.

### 2. Experimental

To a solution of 11*H*-indeno[1,2-*b*]quinoxalin-11-one (0.3 g, 1.29 mmol) and proline (0.208 g, 1.8 mmol) in dry acetonitrile, was added 2,2,7,7-tetramethyl-5-(3-nitro-2*H*-chromen-2-yl)tetrahydro-3aH-bis[1,3] dioxolo[4,5 - b:4',5'-d]pyran (0.52 g, 1.29 mmol) under a nitrogen atmosphere. The reaction mixture was refluxed for 16 h in a Dean-Stark apparatus to give the cycloadduct. After completion of the reaction as indicated by TLC, the solvent was evaporated under reduced pressure. The crude product was extracted with dichloromethane. The organic layer was dried with anhydrous sodium sulfate and concentrated *in vacuo*. Then the crude product was purified by column chromatography using hexane/EtOAc (7:3) as eluent (yield: 0.7 g, 80%). Colourless block shaped crystals were obtained by slow evaporation of a solution of the title compound in ethyl acetate at room temperature.

### 3. Refinement

Hydrogen atoms were positioned geometrically and allowed to ride on their parent atoms, with C—H = 0.93 - 0.97 Å and U_iso_(H) = 1.5 U_eq_(C) for methyl H atoms and 1.2 U_eq_(C) for other H atoms.

### Crystal data

**Table:**

C_39_H_38_N_4_O_8_	*F*(000) = 1456
*M**_r_* = 690.73	*D*_x_ = 1.336 Mg m^−^^3^
Orthorhombic, *P*2_1_2_1_2_1_	Mo *K*α radiation, λ = 0.71073 Å
Hall symbol: P 2ac 2ab	Cell parameters from 8626 reflections
*a* = 11.3150 (9) Å	θ = 1.7–28.4°
*b* = 15.629 (2) Å	µ = 0.09 mm^−^^1^
*c* = 19.419 (2) Å	*T* = 293 K
*V* = 3434.0 (6) Å^3^	Block, colourless
*Z* = 4	0.20 × 0.15 × 0.10 mm

### Data collection

**Table:**

Bruker Kappa APEXII CCD diffractometer	6671 independent reflections
Radiation source: fine-focus sealed tube	3789 reflections with *I* > 2σ(*I*)
Graphite monochromator	*R*_int_ = 0.059
ω and φ scan	θ_max_ = 26.0°, θ_min_ = 1.7°
Absorption correction: multi-scan (*SADABS*; Bruker, 2004)	*h* = −13→13
*T*_min_ = 0.981, *T*_max_ = 0.991	*k* = −19→18
15819 measured reflections	*l* = −23→23

### Refinement

**Table:**

Refinement on *F*^2^	Secondary atom site location: difference Fourier map
Least-squares matrix: full	Hydrogen site location: inferred from neighbouring sites
*R*[*F*^2^ > 2σ(*F*^2^)] = 0.050	H-atom parameters constrained
*wR*(*F*^2^) = 0.124	*w* = 1/[σ^2^(*F*_o_^2^) + (0.047*P*)^2^] where *P* = (*F*_o_^2^ + 2*F*_c_^2^)/3
*S* = 0.97	(Δ/σ)_max_ < 0.001
6671 reflections	Δρ_max_ = 0.17 e Å^−^^3^
460 parameters	Δρ_min_ = −0.20 e Å^−^^3^
0 restraints	Absolute structure: Flack (1983), 2933 Friedel pairs
Primary atom site location: structure-invariant direct methods	Absolute structure parameter: 0.30 (13)

### Special details

**Table:**

Geometry. All e.s.d.'s (except the e.s.d. in the dihedral angle between two l.s. planes) are estimated using the full covariance matrix. The cell e.s.d.'s are taken into account individually in the estimation of e.s.d.'s in distances, angles and torsion angles; correlations between e.s.d.'s in cell parameters are only used when they are defined by crystal symmetry. An approximate (isotropic) treatment of cell e.s.d.'s is used for estimating e.s.d.'s involving l.s. planes.
Refinement. Refinement of *F*^2^ against ALL reflections. The weighted *R*-factor *wR* and goodness of fit *S* are based on *F*^2^, conventional *R*-factors *R* are based on *F*, with *F* set to zero for negative *F*^2^. The threshold expression of *F*^2^ > σ(*F*^2^) is used only for calculating *R*-factors(gt) *etc*. and is not relevant to the choice of reflections for refinement. *R*-factors based on *F*^2^ are statistically about twice as large as those based on *F*, and *R*- factors based on ALL data will be even larger.

### Fractional atomic coordinates and isotropic or equivalent isotropic displacement parameters (Å^2^)

**Table:**

	*x*	*y*	*z*	*U*_iso_*/*U*_eq_	
O1	0.03697 (19)	0.83284 (16)	−0.00002 (10)	0.0524 (6)	
O2	−0.1751 (2)	0.73787 (18)	0.04849 (14)	0.0690 (8)	
O3	−0.3060 (2)	0.8322 (2)	0.07234 (15)	0.0819 (9)	
O4	0.00221 (19)	0.73589 (14)	0.16455 (9)	0.0450 (5)	
O5	−0.0801 (2)	0.60162 (17)	0.15228 (13)	0.0645 (7)	
O6	0.0907 (2)	0.55340 (17)	0.10693 (13)	0.0635 (7)	
O7	0.2843 (2)	0.68893 (18)	0.20258 (12)	0.0672 (7)	
O8	0.23002 (19)	0.81048 (15)	0.14967 (12)	0.0568 (7)	
N1	0.0704 (3)	1.2133 (2)	0.11551 (13)	0.0563 (8)	
N2	0.0858 (2)	1.03075 (19)	0.13143 (14)	0.0522 (7)	
N3	−0.1654 (2)	0.98556 (18)	0.16878 (13)	0.0428 (7)	
N4	−0.2032 (3)	0.8083 (2)	0.06782 (14)	0.0507 (7)	
C1	−0.2053 (3)	1.1070 (2)	0.08517 (16)	0.0484 (9)	
C2	−0.3219 (3)	1.1091 (3)	0.06145 (19)	0.0643 (11)	
H2	−0.3667	1.0593	0.0587	0.077*	
C3	−0.3687 (4)	1.1872 (3)	0.0422 (2)	0.0722 (12)	
H3	−0.4460	1.1893	0.0261	0.087*	
C4	−0.3057 (4)	1.2611 (3)	0.0460 (2)	0.0744 (12)	
H4	−0.3407	1.3127	0.0337	0.089*	
C5	−0.1888 (4)	1.2597 (3)	0.06840 (18)	0.0631 (10)	
H5	−0.1443	1.3096	0.0705	0.076*	
C6	−0.1408 (3)	1.1816 (2)	0.08745 (15)	0.0485 (9)	
C7	−0.0190 (3)	1.1607 (2)	0.10770 (15)	0.0450 (8)	
C8	0.1751 (3)	1.1725 (3)	0.13211 (17)	0.0559 (10)	
C9	0.2774 (4)	1.2228 (3)	0.1405 (2)	0.0753 (13)	
H9	0.2739	1.2818	0.1346	0.090*	
C10	0.3812 (4)	1.1847 (4)	0.1572 (2)	0.0878 (15)	
H10	0.4482	1.2182	0.1637	0.105*	
C11	0.3893 (4)	1.0961 (4)	0.1649 (3)	0.0977 (16)	
H11	0.4614	1.0712	0.1760	0.117*	
C12	0.2919 (3)	1.0457 (3)	0.1562 (2)	0.0832 (13)	
H12	0.2977	0.9867	0.1610	0.100*	
C13	0.1827 (3)	1.0838 (3)	0.13974 (18)	0.0556 (10)	
C14	−0.0112 (3)	1.0707 (2)	0.11541 (15)	0.0438 (8)	
C15	−0.1313 (3)	1.0280 (2)	0.10405 (15)	0.0428 (8)	
C16	−0.2921 (3)	0.9806 (3)	0.18833 (17)	0.0554 (10)	
H16A	−0.3413	0.9653	0.1493	0.067*	
H16B	−0.3198	1.0341	0.2078	0.067*	
C17	−0.2893 (3)	0.9102 (3)	0.24158 (18)	0.0608 (10)	
H17A	−0.3663	0.8834	0.2457	0.073*	
H17B	−0.2664	0.9327	0.2862	0.073*	
C18	−0.1979 (3)	0.8461 (3)	0.21548 (16)	0.0575 (10)	
H18A	−0.2359	0.7992	0.1914	0.069*	
H18B	−0.1520	0.8230	0.2534	0.069*	
C19	−0.1189 (3)	0.8974 (2)	0.16643 (14)	0.0410 (8)	
H19	−0.0391	0.8983	0.1859	0.049*	
C20	−0.1080 (3)	0.8724 (2)	0.08846 (15)	0.0402 (8)	
C21	0.0170 (3)	0.8386 (2)	0.07265 (14)	0.0385 (7)	
H21	0.0735	0.8803	0.0911	0.046*	
C22	0.0241 (3)	0.9087 (2)	−0.03512 (15)	0.0440 (8)	
C23	0.0911 (3)	0.9177 (3)	−0.09451 (17)	0.0596 (10)	
H23	0.1434	0.8749	−0.1078	0.071*	
C24	0.0793 (4)	0.9897 (3)	−0.13301 (18)	0.0666 (11)	
H24	0.1243	0.9965	−0.1727	0.080*	
C25	0.0006 (4)	1.0533 (3)	−0.11346 (18)	0.0685 (11)	
H25	−0.0068	1.1026	−0.1399	0.082*	
C26	−0.0667 (3)	1.0433 (2)	−0.05465 (16)	0.0565 (10)	
H26	−0.1201	1.0857	−0.0421	0.068*	
C27	−0.0553 (3)	0.9703 (2)	−0.01397 (14)	0.0426 (8)	
C28	−0.1323 (3)	0.9566 (2)	0.04885 (15)	0.0426 (8)	
H28	−0.2138	0.9528	0.0320	0.051*	
C29	0.0536 (3)	0.7508 (2)	0.09899 (15)	0.0410 (8)	
H29	0.0258	0.7070	0.0667	0.049*	
C30	0.0092 (3)	0.6504 (2)	0.18407 (17)	0.0519 (9)	
H30	−0.0012	0.6468	0.2341	0.062*	
C31	−0.0300 (3)	0.5300 (3)	0.1170 (2)	0.0649 (11)	
C32	0.1236 (3)	0.6036 (2)	0.16469 (18)	0.0553 (9)	
H32	0.1489	0.5665	0.2026	0.066*	
C33	0.2250 (3)	0.6609 (2)	0.14226 (18)	0.0516 (9)	
H33	0.2796	0.6288	0.1129	0.062*	
C34	0.3182 (3)	0.7751 (3)	0.19214 (18)	0.0590 (10)	
C35	0.1876 (3)	0.7440 (2)	0.10601 (16)	0.0445 (8)	
H35	0.2251	0.7479	0.0606	0.053*	
C36	−0.0875 (4)	0.5211 (3)	0.0485 (2)	0.0846 (13)	
H36A	−0.1688	0.5053	0.0545	0.127*	
H36B	−0.0476	0.4776	0.0224	0.127*	
H36C	−0.0831	0.5745	0.0243	0.127*	
C37	−0.0398 (4)	0.4515 (3)	0.1621 (2)	0.0955 (15)	
H37A	−0.1215	0.4370	0.1681	0.143*	
H37B	−0.0048	0.4631	0.2061	0.143*	
H37C	0.0008	0.4047	0.1406	0.143*	
C38	0.4382 (3)	0.7814 (3)	0.1578 (2)	0.0901 (15)	
H38A	0.4587	0.8405	0.1517	0.135*	
H38B	0.4354	0.7536	0.1137	0.135*	
H38C	0.4965	0.7541	0.1862	0.135*	
C39	0.3150 (4)	0.8192 (3)	0.2610 (2)	0.0891 (15)	
H39A	0.3377	0.8780	0.2555	0.134*	
H39B	0.3689	0.7914	0.2920	0.134*	
H39C	0.2364	0.8164	0.2795	0.134*	

### Atomic displacement parameters (Å^2^)

**Table:**

	*U*^11^	*U*^22^	*U*^33^	*U*^12^	*U*^13^	*U*^23^
O1	0.0671 (15)	0.0485 (16)	0.0417 (12)	0.0044 (13)	0.0116 (12)	0.0053 (11)
O2	0.0695 (17)	0.0440 (18)	0.093 (2)	−0.0040 (14)	−0.0303 (15)	−0.0075 (15)
O3	0.0404 (15)	0.086 (2)	0.120 (2)	−0.0129 (15)	0.0069 (15)	−0.0265 (18)
O4	0.0532 (13)	0.0413 (14)	0.0403 (11)	0.0031 (11)	0.0078 (11)	0.0071 (10)
O5	0.0573 (15)	0.0536 (18)	0.0828 (17)	−0.0092 (14)	0.0014 (14)	−0.0015 (14)
O6	0.0593 (16)	0.0528 (17)	0.0785 (17)	−0.0045 (13)	−0.0014 (14)	−0.0130 (13)
O7	0.0681 (17)	0.061 (2)	0.0721 (17)	−0.0002 (15)	−0.0298 (14)	0.0014 (14)
O8	0.0506 (14)	0.0506 (16)	0.0693 (15)	−0.0014 (12)	−0.0141 (12)	−0.0111 (13)
N1	0.061 (2)	0.053 (2)	0.0542 (18)	−0.0164 (17)	0.0069 (15)	−0.0056 (15)
N2	0.0402 (16)	0.054 (2)	0.0624 (18)	−0.0037 (16)	−0.0054 (14)	−0.0007 (15)
N3	0.0354 (15)	0.0474 (19)	0.0456 (15)	−0.0028 (13)	0.0002 (12)	−0.0001 (14)
N4	0.053 (2)	0.053 (2)	0.0467 (16)	−0.0075 (17)	−0.0057 (15)	0.0020 (15)
C1	0.047 (2)	0.053 (2)	0.0453 (19)	0.0040 (19)	−0.0004 (17)	0.0011 (18)
C2	0.053 (2)	0.067 (3)	0.073 (2)	0.006 (2)	−0.012 (2)	0.005 (2)
C3	0.064 (2)	0.082 (4)	0.071 (3)	0.020 (3)	−0.010 (2)	0.004 (3)
C4	0.085 (3)	0.068 (3)	0.071 (3)	0.031 (3)	−0.005 (2)	0.001 (2)
C5	0.077 (3)	0.046 (2)	0.066 (2)	0.010 (2)	0.000 (2)	0.0013 (19)
C6	0.058 (2)	0.045 (2)	0.0419 (19)	0.0076 (19)	0.0032 (16)	−0.0001 (17)
C7	0.053 (2)	0.042 (2)	0.0400 (17)	−0.0013 (19)	0.0053 (16)	−0.0043 (16)
C8	0.050 (2)	0.067 (3)	0.051 (2)	−0.014 (2)	0.0076 (18)	−0.010 (2)
C9	0.064 (3)	0.083 (3)	0.079 (3)	−0.029 (3)	0.015 (2)	−0.017 (2)
C10	0.053 (3)	0.112 (5)	0.099 (3)	−0.026 (3)	0.004 (2)	−0.019 (3)
C11	0.041 (3)	0.125 (5)	0.126 (4)	−0.005 (3)	−0.010 (3)	−0.006 (4)
C12	0.043 (2)	0.088 (3)	0.119 (4)	−0.001 (2)	−0.011 (2)	−0.001 (3)
C13	0.045 (2)	0.063 (3)	0.058 (2)	−0.009 (2)	−0.0004 (18)	−0.0048 (19)
C14	0.045 (2)	0.046 (2)	0.0395 (18)	−0.0041 (17)	0.0020 (16)	−0.0048 (16)
C15	0.0364 (17)	0.045 (2)	0.0472 (19)	−0.0039 (16)	−0.0029 (15)	0.0032 (17)
C16	0.0368 (19)	0.070 (3)	0.060 (2)	−0.0058 (19)	0.0071 (17)	−0.007 (2)
C17	0.051 (2)	0.074 (3)	0.056 (2)	−0.008 (2)	0.0116 (19)	−0.002 (2)
C18	0.065 (2)	0.063 (3)	0.0441 (19)	−0.006 (2)	0.0124 (18)	−0.0006 (18)
C19	0.0402 (18)	0.045 (2)	0.0373 (16)	−0.0035 (16)	0.0006 (15)	−0.0018 (15)
C20	0.0385 (18)	0.041 (2)	0.0415 (17)	−0.0098 (15)	0.0005 (15)	−0.0001 (15)
C21	0.0418 (18)	0.039 (2)	0.0352 (16)	−0.0026 (16)	0.0034 (14)	0.0011 (15)
C22	0.051 (2)	0.044 (2)	0.0359 (17)	−0.0040 (18)	−0.0014 (17)	0.0072 (16)
C23	0.061 (2)	0.066 (3)	0.052 (2)	0.001 (2)	0.0160 (19)	0.006 (2)
C24	0.077 (3)	0.073 (3)	0.050 (2)	−0.006 (2)	0.013 (2)	0.018 (2)
C25	0.084 (3)	0.069 (3)	0.052 (2)	−0.007 (2)	−0.002 (2)	0.023 (2)
C26	0.062 (2)	0.058 (3)	0.050 (2)	0.002 (2)	−0.0056 (19)	0.0097 (19)
C27	0.0442 (18)	0.048 (2)	0.0358 (17)	−0.0064 (18)	−0.0048 (15)	0.0047 (16)
C28	0.0380 (17)	0.045 (2)	0.0453 (18)	−0.0051 (16)	−0.0056 (15)	0.0019 (16)
C29	0.0442 (19)	0.041 (2)	0.0376 (17)	0.0016 (16)	0.0054 (15)	−0.0015 (15)
C30	0.058 (2)	0.053 (2)	0.0449 (18)	−0.005 (2)	−0.0010 (18)	0.0054 (17)
C31	0.059 (2)	0.048 (3)	0.088 (3)	−0.004 (2)	−0.006 (2)	0.000 (2)
C32	0.060 (2)	0.047 (2)	0.059 (2)	0.0036 (19)	−0.0098 (19)	0.002 (2)
C33	0.0454 (19)	0.050 (2)	0.059 (2)	0.0082 (18)	−0.0054 (18)	−0.0081 (19)
C34	0.051 (2)	0.063 (3)	0.064 (2)	0.009 (2)	−0.0115 (19)	−0.010 (2)
C35	0.0448 (19)	0.047 (2)	0.0415 (17)	−0.0003 (17)	0.0038 (15)	−0.0055 (17)
C36	0.077 (3)	0.085 (3)	0.092 (3)	−0.010 (3)	−0.017 (3)	−0.013 (3)
C37	0.102 (4)	0.059 (3)	0.126 (4)	−0.014 (3)	−0.013 (3)	0.011 (3)
C38	0.051 (3)	0.101 (4)	0.119 (4)	−0.001 (2)	−0.002 (3)	−0.020 (3)
C39	0.098 (3)	0.094 (4)	0.076 (3)	0.012 (3)	−0.028 (3)	−0.025 (3)

### Geometric parameters (Å, º)

**Table:**

O1—C22	1.376 (4)	C17—C18	1.527 (5)
O1—C21	1.432 (3)	C17—H17A	0.9700
O2—N4	1.205 (4)	C17—H17B	0.9700
O3—N4	1.225 (4)	C18—C19	1.533 (4)
O4—C30	1.391 (4)	C18—H18A	0.9700
O4—C29	1.419 (3)	C18—H18B	0.9700
O5—C30	1.408 (4)	C19—C20	1.569 (4)
O5—C31	1.429 (5)	C19—H19	0.9800
O6—C32	1.419 (4)	C20—C21	1.541 (4)
O6—C31	1.427 (4)	C20—C28	1.549 (4)
O7—C34	1.414 (5)	C21—C29	1.522 (4)
O7—C33	1.419 (4)	C21—H21	0.9800
O8—C34	1.408 (4)	C22—C27	1.379 (4)
O8—C35	1.424 (4)	C22—C23	1.387 (4)
N1—C7	1.313 (4)	C23—C24	1.358 (5)
N1—C8	1.383 (5)	C23—H23	0.9300
N2—C14	1.301 (4)	C24—C25	1.386 (6)
N2—C13	1.384 (4)	C24—H24	0.9300
N3—C15	1.473 (4)	C25—C26	1.381 (5)
N3—C19	1.475 (4)	C25—H25	0.9300
N3—C16	1.486 (4)	C26—C27	1.394 (5)
N4—C20	1.524 (4)	C26—H26	0.9300
C1—C6	1.376 (5)	C27—C28	1.514 (4)
C1—C2	1.397 (5)	C28—H28	0.9800
C1—C15	1.537 (5)	C29—C35	1.526 (4)
C2—C3	1.382 (5)	C29—H29	0.9800
C2—H2	0.9300	C30—C32	1.534 (5)
C3—C4	1.359 (6)	C30—H30	0.9800
C3—H3	0.9300	C31—C36	1.488 (5)
C4—C5	1.392 (6)	C31—C37	1.510 (5)
C4—H4	0.9300	C32—C33	1.519 (5)
C5—C6	1.386 (5)	C32—H32	0.9800
C5—H5	0.9300	C33—C35	1.537 (5)
C6—C7	1.470 (5)	C33—H33	0.9800
C7—C14	1.416 (5)	C34—C39	1.506 (5)
C8—C13	1.398 (5)	C34—C38	1.516 (5)
C8—C9	1.409 (5)	C35—H35	0.9800
C9—C10	1.356 (6)	C36—H36A	0.9600
C9—H9	0.9300	C36—H36B	0.9600
C10—C11	1.396 (7)	C36—H36C	0.9600
C10—H10	0.9300	C37—H37A	0.9600
C11—C12	1.364 (6)	C37—H37B	0.9600
C11—H11	0.9300	C37—H37C	0.9600
C12—C13	1.408 (5)	C38—H38A	0.9600
C12—H12	0.9300	C38—H38B	0.9600
C14—C15	1.530 (4)	C38—H38C	0.9600
C15—C28	1.547 (4)	C39—H39A	0.9600
C16—C17	1.511 (5)	C39—H39B	0.9600
C16—H16A	0.9700	C39—H39C	0.9600
C16—H16B	0.9700		
			
C22—O1—C21	114.7 (2)	C29—C21—C20	119.5 (2)
C30—O4—C29	112.3 (2)	O1—C21—H21	107.4
C30—O5—C31	110.5 (3)	C29—C21—H21	107.4
C32—O6—C31	106.5 (3)	C20—C21—H21	107.4
C34—O7—C33	107.7 (3)	O1—C22—C27	121.5 (3)
C34—O8—C35	107.5 (3)	O1—C22—C23	116.2 (3)
C7—N1—C8	113.4 (3)	C27—C22—C23	122.2 (3)
C14—N2—C13	114.1 (3)	C24—C23—C22	119.2 (4)
C15—N3—C19	107.5 (2)	C24—C23—H23	120.4
C15—N3—C16	119.6 (2)	C22—C23—H23	120.4
C19—N3—C16	107.6 (3)	C23—C24—C25	120.4 (3)
O2—N4—O3	123.4 (3)	C23—C24—H24	119.8
O2—N4—C20	119.7 (3)	C25—C24—H24	119.8
O3—N4—C20	116.9 (3)	C26—C25—C24	120.0 (4)
C6—C1—C2	119.4 (3)	C26—C25—H25	120.0
C6—C1—C15	112.6 (3)	C24—C25—H25	120.0
C2—C1—C15	127.7 (3)	C25—C26—C27	120.7 (4)
C3—C2—C1	118.1 (4)	C25—C26—H26	119.7
C3—C2—H2	120.9	C27—C26—H26	119.7
C1—C2—H2	120.9	C22—C27—C26	117.6 (3)
C4—C3—C2	122.3 (4)	C22—C27—C28	121.0 (3)
C4—C3—H3	118.8	C26—C27—C28	121.3 (3)
C2—C3—H3	118.8	C27—C28—C15	116.9 (3)
C3—C4—C5	120.1 (4)	C27—C28—C20	114.7 (3)
C3—C4—H4	120.0	C15—C28—C20	105.5 (2)
C5—C4—H4	120.0	C27—C28—H28	106.3
C6—C5—C4	118.0 (4)	C15—C28—H28	106.3
C6—C5—H5	121.0	C20—C28—H28	106.3
C4—C5—H5	121.0	O4—C29—C21	109.8 (2)
C1—C6—C5	122.0 (3)	O4—C29—C35	108.4 (2)
C1—C6—C7	108.5 (3)	C21—C29—C35	111.3 (3)
C5—C6—C7	129.4 (4)	O4—C29—H29	109.1
N1—C7—C14	124.2 (3)	C21—C29—H29	109.1
N1—C7—C6	127.8 (3)	C35—C29—H29	109.1
C14—C7—C6	107.9 (3)	O4—C30—O5	111.1 (3)
N1—C8—C13	122.3 (3)	O4—C30—C32	116.0 (3)
N1—C8—C9	118.3 (4)	O5—C30—C32	103.9 (3)
C13—C8—C9	119.4 (4)	O4—C30—H30	108.5
C10—C9—C8	119.6 (5)	O5—C30—H30	108.5
C10—C9—H9	120.2	C32—C30—H30	108.5
C8—C9—H9	120.2	O6—C31—O5	104.2 (3)
C9—C10—C11	121.3 (4)	O6—C31—C36	108.6 (3)
C9—C10—H10	119.4	O5—C31—C36	109.2 (3)
C11—C10—H10	119.4	O6—C31—C37	110.9 (3)
C12—C11—C10	120.4 (4)	O5—C31—C37	109.2 (3)
C12—C11—H11	119.8	C36—C31—C37	114.2 (4)
C10—C11—H11	119.8	O6—C32—C33	107.3 (3)
C11—C12—C13	119.5 (5)	O6—C32—C30	103.6 (3)
C11—C12—H12	120.2	C33—C32—C30	115.3 (3)
C13—C12—H12	120.2	O6—C32—H32	110.1
N2—C13—C8	122.2 (3)	C33—C32—H32	110.1
N2—C13—C12	117.9 (4)	C30—C32—H32	110.1
C8—C13—C12	119.8 (4)	O7—C33—C32	107.6 (3)
N2—C14—C7	123.7 (3)	O7—C33—C35	104.3 (3)
N2—C14—C15	125.0 (3)	C32—C33—C35	115.0 (3)
C7—C14—C15	111.3 (3)	O7—C33—H33	109.9
N3—C15—C14	107.8 (2)	C32—C33—H33	109.9
N3—C15—C1	115.0 (3)	C35—C33—H33	109.9
C14—C15—C1	99.6 (3)	O8—C34—O7	105.4 (3)
N3—C15—C28	105.4 (3)	O8—C34—C39	108.9 (3)
C14—C15—C28	114.9 (3)	O7—C34—C39	107.6 (3)
C1—C15—C28	114.2 (2)	O8—C34—C38	110.6 (3)
N3—C16—C17	101.1 (3)	O7—C34—C38	111.6 (3)
N3—C16—H16A	111.5	C39—C34—C38	112.5 (4)
C17—C16—H16A	111.5	O8—C35—C29	109.7 (3)
N3—C16—H16B	111.5	O8—C35—C33	104.5 (2)
C17—C16—H16B	111.5	C29—C35—C33	111.9 (3)
H16A—C16—H16B	109.4	O8—C35—H35	110.2
C16—C17—C18	105.4 (3)	C29—C35—H35	110.2
C16—C17—H17A	110.7	C33—C35—H35	110.2
C18—C17—H17A	110.7	C31—C36—H36A	109.5
C16—C17—H17B	110.7	C31—C36—H36B	109.5
C18—C17—H17B	110.7	H36A—C36—H36B	109.5
H17A—C17—H17B	108.8	C31—C36—H36C	109.5
C17—C18—C19	104.9 (3)	H36A—C36—H36C	109.5
C17—C18—H18A	110.8	H36B—C36—H36C	109.5
C19—C18—H18A	110.8	C31—C37—H37A	109.5
C17—C18—H18B	110.8	C31—C37—H37B	109.5
C19—C18—H18B	110.8	H37A—C37—H37B	109.5
H18A—C18—H18B	108.8	C31—C37—H37C	109.5
N3—C19—C18	105.2 (3)	H37A—C37—H37C	109.5
N3—C19—C20	106.9 (2)	H37B—C37—H37C	109.5
C18—C19—C20	121.0 (3)	C34—C38—H38A	109.5
N3—C19—H19	107.7	C34—C38—H38B	109.5
C18—C19—H19	107.7	H38A—C38—H38B	109.5
C20—C19—H19	107.7	C34—C38—H38C	109.5
N4—C20—C21	111.8 (3)	H38A—C38—H38C	109.5
N4—C20—C28	107.6 (2)	H38B—C38—H38C	109.5
C21—C20—C28	110.8 (2)	C34—C39—H39A	109.5
N4—C20—C19	111.2 (2)	C34—C39—H39B	109.5
C21—C20—C19	110.5 (2)	H39A—C39—H39B	109.5
C28—C20—C19	104.7 (2)	C34—C39—H39C	109.5
O1—C21—C29	103.4 (2)	H39A—C39—H39C	109.5
O1—C21—C20	111.3 (2)	H39B—C39—H39C	109.5
			
C6—C1—C2—C3	−1.2 (5)	N4—C20—C21—O1	68.0 (3)
C15—C1—C2—C3	−174.9 (3)	C28—C20—C21—O1	−52.0 (3)
C1—C2—C3—C4	−0.4 (6)	C19—C20—C21—O1	−167.5 (2)
C2—C3—C4—C5	1.6 (6)	N4—C20—C21—C29	−52.3 (3)
C3—C4—C5—C6	−1.1 (6)	C28—C20—C21—C29	−172.3 (3)
C2—C1—C6—C5	1.6 (5)	C19—C20—C21—C29	72.1 (3)
C15—C1—C6—C5	176.2 (3)	C21—O1—C22—C27	−31.8 (4)
C2—C1—C6—C7	−174.6 (3)	C21—O1—C22—C23	151.3 (3)
C15—C1—C6—C7	0.0 (4)	O1—C22—C23—C24	177.6 (3)
C4—C5—C6—C1	−0.4 (5)	C27—C22—C23—C24	0.7 (5)
C4—C5—C6—C7	174.9 (3)	C22—C23—C24—C25	−0.5 (6)
C8—N1—C7—C14	0.1 (4)	C23—C24—C25—C26	−0.2 (6)
C8—N1—C7—C6	−177.3 (3)	C24—C25—C26—C27	0.9 (6)
C1—C6—C7—N1	179.6 (3)	O1—C22—C27—C26	−176.8 (3)
C5—C6—C7—N1	3.8 (6)	C23—C22—C27—C26	0.0 (5)
C1—C6—C7—C14	1.8 (4)	O1—C22—C27—C28	−0.3 (5)
C5—C6—C7—C14	−174.0 (3)	C23—C22—C27—C28	176.5 (3)
C7—N1—C8—C13	−0.2 (5)	C25—C26—C27—C22	−0.7 (5)
C7—N1—C8—C9	178.9 (3)	C25—C26—C27—C28	−177.2 (3)
N1—C8—C9—C10	179.5 (4)	C22—C27—C28—C15	128.1 (3)
C13—C8—C9—C10	−1.4 (6)	C26—C27—C28—C15	−55.5 (4)
C8—C9—C10—C11	1.4 (7)	C22—C27—C28—C20	3.8 (4)
C9—C10—C11—C12	−0.5 (8)	C26—C27—C28—C20	−179.8 (3)
C10—C11—C12—C13	−0.4 (8)	N3—C15—C28—C27	−155.5 (3)
C14—N2—C13—C8	0.6 (5)	C14—C15—C28—C27	−36.9 (4)
C14—N2—C13—C12	−179.1 (3)	C1—C15—C28—C27	77.4 (3)
N1—C8—C13—N2	−0.2 (5)	N3—C15—C28—C20	−26.6 (3)
C9—C8—C13—N2	−179.2 (3)	C14—C15—C28—C20	91.9 (3)
N1—C8—C13—C12	179.5 (3)	C1—C15—C28—C20	−153.8 (3)
C9—C8—C13—C12	0.5 (5)	N4—C20—C28—C27	−100.7 (3)
C11—C12—C13—N2	−179.8 (4)	C21—C20—C28—C27	21.8 (3)
C11—C12—C13—C8	0.4 (6)	C19—C20—C28—C27	140.9 (3)
C13—N2—C14—C7	−0.7 (5)	N4—C20—C28—C15	129.2 (3)
C13—N2—C14—C15	−179.3 (3)	C21—C20—C28—C15	−108.3 (3)
N1—C7—C14—N2	0.3 (5)	C19—C20—C28—C15	10.8 (3)
C6—C7—C14—N2	178.2 (3)	C30—O4—C29—C21	165.9 (3)
N1—C7—C14—C15	179.1 (3)	C30—O4—C29—C35	−72.3 (3)
C6—C7—C14—C15	−3.0 (4)	O1—C21—C29—O4	−160.0 (2)
C19—N3—C15—C14	−90.3 (3)	C20—C21—C29—O4	−35.7 (4)
C16—N3—C15—C14	146.7 (3)	O1—C21—C29—C35	80.0 (3)
C19—N3—C15—C1	159.6 (3)	C20—C21—C29—C35	−155.7 (3)
C16—N3—C15—C1	36.6 (4)	C29—O4—C30—O5	−80.2 (3)
C19—N3—C15—C28	32.9 (3)	C29—O4—C30—C32	38.1 (4)
C16—N3—C15—C28	−90.1 (3)	C31—O5—C30—O4	126.4 (3)
N2—C14—C15—N3	61.3 (4)	C31—O5—C30—C32	0.9 (4)
C7—C14—C15—N3	−117.5 (3)	C32—O6—C31—O5	33.2 (4)
N2—C14—C15—C1	−178.4 (3)	C32—O6—C31—C36	149.5 (3)
C7—C14—C15—C1	2.8 (3)	C32—O6—C31—C37	−84.2 (4)
N2—C14—C15—C28	−55.9 (4)	C30—O5—C31—O6	−20.6 (4)
C7—C14—C15—C28	125.4 (3)	C30—O5—C31—C36	−136.5 (3)
C6—C1—C15—N3	113.3 (3)	C30—O5—C31—C37	98.0 (4)
C2—C1—C15—N3	−72.6 (4)	C31—O6—C32—C33	−154.8 (3)
C6—C1—C15—C14	−1.7 (3)	C31—O6—C32—C30	−32.4 (3)
C2—C1—C15—C14	172.4 (3)	O4—C30—C32—O6	−103.1 (3)
C6—C1—C15—C28	−124.7 (3)	O5—C30—C32—O6	19.1 (3)
C2—C1—C15—C28	49.4 (4)	O4—C30—C32—C33	13.9 (4)
C15—N3—C16—C17	162.1 (3)	O5—C30—C32—C33	136.1 (3)
C19—N3—C16—C17	39.1 (3)	C34—O7—C33—C32	−142.0 (3)
N3—C16—C17—C18	−37.0 (3)	C34—O7—C33—C35	−19.4 (3)
C16—C17—C18—C19	22.1 (4)	O6—C32—C33—O7	−160.4 (3)
C15—N3—C19—C18	−155.9 (2)	C30—C32—C33—O7	84.7 (4)
C16—N3—C19—C18	−25.8 (3)	O6—C32—C33—C35	83.9 (4)
C15—N3—C19—C20	−26.1 (3)	C30—C32—C33—C35	−31.0 (4)
C16—N3—C19—C20	104.1 (3)	C35—O8—C34—O7	−31.9 (3)
C17—C18—C19—N3	1.7 (3)	C35—O8—C34—C39	−147.1 (3)
C17—C18—C19—C20	−119.3 (3)	C35—O8—C34—C38	88.8 (4)
O2—N4—C20—C21	6.9 (4)	C33—O7—C34—O8	32.1 (3)
O3—N4—C20—C21	−173.4 (3)	C33—O7—C34—C39	148.2 (3)
O2—N4—C20—C28	128.7 (3)	C33—O7—C34—C38	−88.0 (3)
O3—N4—C20—C28	−51.6 (4)	C34—O8—C35—C29	139.5 (3)
O2—N4—C20—C19	−117.2 (3)	C34—O8—C35—C33	19.3 (3)
O3—N4—C20—C19	62.5 (4)	O4—C29—C35—O8	−64.6 (3)
N3—C19—C20—N4	−107.3 (3)	C21—C29—C35—O8	56.2 (3)
C18—C19—C20—N4	12.9 (4)	O4—C29—C35—C33	50.9 (4)
N3—C19—C20—C21	128.0 (3)	C21—C29—C35—C33	171.7 (2)
C18—C19—C20—C21	−111.9 (3)	O7—C33—C35—O8	0.1 (3)
N3—C19—C20—C28	8.6 (3)	C32—C33—C35—O8	117.7 (3)
C18—C19—C20—C28	128.8 (3)	O7—C33—C35—C29	−118.6 (3)
C22—O1—C21—C29	−172.3 (2)	C32—C33—C35—C29	−1.0 (4)
C22—O1—C21—C20	58.3 (3)		

### Hydrogen-bond geometry (Å, º)

Cg8 is the centroid of the N1/C7/C14/N2/C13/C8 ring.

**Table:**

*D*—H···*A*	*D*—H	H···*A*	*D*···*A*	*D*—H···*A*
C35—H35···O2^i^	0.98	2.41	3.390 (4)	177
C16—H16*A*···O3	0.97	2.59	3.237 (5)	124
C21—H21···N2	0.98	2.48	3.305 (4)	141
C30—H30···*Cg*8^ii^	0.98	2.93	3.895 (4)	168

Symmetry codes: (i) *x*+1/2, −*y*+3/2, −*z*; (ii) −*x*, *y*−1/2, −*z*+1/2.
